# Experience in Multiple Sclerosis Patients with COVID-19 and Disease-Modifying Therapies: A Review of 873 Published Cases

**DOI:** 10.3390/jcm9124067

**Published:** 2020-12-16

**Authors:** Nora Möhn, Franz F. Konen, Refik Pul, Christoph Kleinschnitz, Harald Prüss, Torsten Witte, Martin Stangel, Thomas Skripuletz

**Affiliations:** 1 Department of Neurology, Hanover Medical School, 30625 Hannover, Germany; moehn.nora@mh-hannover.de (N.M.); konen.felix@mh-hannover.de (F.F.K.); stangel.martin@mh-hannover.de (M.S.); 2 Department of Neurology, University Hospital Essen, 45147 Essen, Germany; refik.pul@uk-essen.de (R.P.); christoph.kleinschnitz@uk-essen.de (C.K.); 3 Department of Neurology and Experimental Neurology, Charité Universitätsmedizin Berlin and German Center for Neurodegenerative Diseases (DZNE), 10117 Berlin, Germany; harald.pruess@dzne.de; 4 Department of Rheumatology & Immunology, Hannover Medical School, 30625 Hannover, Germany; witte.torsten@mh-hannover.de

**Keywords:** SARS-CoV-2 pandemic, COVID-19, multiple sclerosis, disease-modifying therapies

## Abstract

The severe acute respiratory syndrome coronavirus-2 (SARS-CoV-2) pandemic is a challenge for all participants in the healthcare system. At the beginning of the pandemic, many physicians asked themselves what risk their patients, especially those with chronic diseases, were exposed to. We present an overview of all patients with multiple sclerosis (MS) and SARS-CoV-2 infection published in the literature so far. In total, there are publications on 873 SARS-CoV-2 positive MS patients and information on the outcome can be given for 700 patients. With regard to the different disease modifying therapies (DMTs), by far the most cases were described under anti-CD20 treatment (*n* = 317). The mortality rate of all MS patients was 4% and a further 3% required invasive or non-invasive ventilation. When looking at the severe and fatal cases, it is particularly noticeable that patients without DMTs, with previous cardiovascular diseases, or with a severe degree of disability are at risk. Immunosuppressive therapy itself does not appear to be a substantial risk factor. Rather, it is reasonable to assume that the therapies could be protective, either directly, by mitigating the cytokine storm, or indirectly, by reducing the disease activity of MS.

## 1. Introduction

The outbreak of the novel coronavirus, named severe acute respiratory syndrome coronavirus 2 (SARS-CoV-2), and the resulting global pandemic has confronted the world and its health authorities with one of the biggest challenges in recent history. The rapid disease spread is especially impressive, as the virus has reached every country worldwide in less than six months [1]. SARS-CoV-2 is known to primarily affect the patients’ lungs [2,3]. The primary cause of SARS-CoV-2 mortality is acute respiratory distress syndrome (ARDS) initiated by epithelial infection and alveolar macrophage activation in the lungs. Typical clinical presentations are fever, dry cough, fatigue, ageusia or anosmia and, less often, symptoms of sputum production, headache, sore throat, or myalgia [4]. However, clinicians and researchers worldwide have reported relevant effects of COVID-19 on other major organs, including blood vessels, brain, gastrointestinal tract, kidney, heart, and liver. In severe COVID-19 infections, the dysregulated immune system responds by secreting cytokines in an uncontrolled manner known as “cytokine storm” syndrome [5,6]. SARS-CoV-2-infected monocytes, macrophages, dendritic cells, and lymphocytes appear to play an essential role in the development and degree of the cytokine storm that, as a consequence, may lead to [7,8,9] leukocyte recruitment to multiple body organs, most notably to the lung parenchyma cells. Such cytokine storms have been noticed earlier in other viral illnesses such as SARS, MERS, or influenza.

One question is crucial for physicians of all specialties, namely which of their patients are particularly at risk during the pandemic? It has been shown that older age and obesity are the most important conditions that increase the mortality risk of SARS-CoV-2 patients [10]. According to the Center for Disease Control and Prevention, patients with asthma, chronic lung disease, diabetes, serious heart conditions, chronic kidney disease, residents of nursing homes or long-term care facilities, and immune-compromised patients may be at a high risk for a severe disease course of COVID-19 [11].

The potential risk of patients with chronic autoimmune diseases, such as multiple sclerosis (MS), could not be fully clarified so far. MS is an immune-mediated central nervous system (CNS) disorder that requires immunosuppressive or immunomodulating disease-modifying therapies (DMTs). The inclusion of immunocompromised patients in the ‘high-risk population’ for COVID-19 is intuitive, as immunosuppression should make a person more likely to contract an infection and may complicate the disease course. However, analyses of large Chinese or Italian cohorts did not identify immunosuppression as a risk factor for disease severity in COVID-19 [12,13]. In addition, data on prior related coronavirus outbreaks in MERS and SARS did not show any evidence of increased risk of infection or morbidity in immunocompromised populations [14,15,16]. There is even the assumption that immunomodulatory therapies might be protective in case of a SARS-CoV-2 infection, since a potentially fatal cytokine storm can be mitigated [17].

A large number of single case reports and case series have been published on patients with MS and SARS-CoV-2 infection, however, an overarching overview is lacking. Here we review the results of all MS patients with SARS-CoV-2 infection (*n* = 873) published in the literature so far. Besides detailed case reports, larger case series or studies were also considered. The outcome of patients is presented in relation to their respective DMTs.

## 2. Materials & Methods

On 22 October 2020 we conducted the literature search via pubmed_ National Center for Biotechnology Information NCBI using the following search terms: COVID-19_multiple sclerosis; coronavirus_multiple sclerosis; sars_multiple sclerosis. The best search term with 279 hits turned out to be coronavirus_multiple sclerosis. Finally, all case reports as well as all case series and larger studies which contained information on the different DMTs and the outcome of the respective patients were included [18,19,20,21,22,23,24,25,26,27,28,29,30,31,32,33,34,35,36,37,38,39,40,41,42,43,44,45,46,47,48,49,50,51,52,53,54,55,56,57,58,59,60,61,62,63,64,65,66,67,68,69,70,71].

## 3. Results

### 3.1. Distribution of DMTs among Published SARS-CoV-2 Positive MS Patients

**Figure 1:**
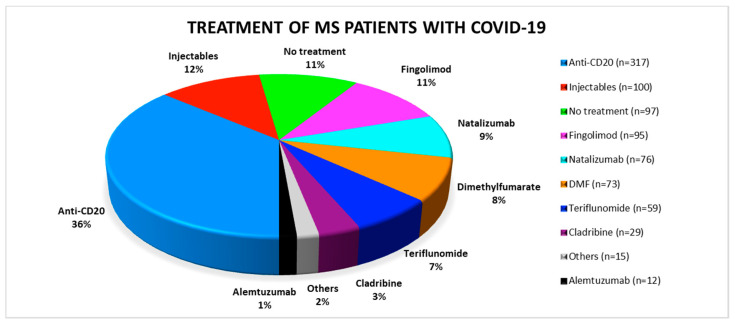
Distribution of the respective disease modifying therapies among the published SARS-CoV-2 positive multiple sclerosis patients. COVID-19: coronavirus disease 2019; DMF: dimethyl-fumarate; MS: multiple sclerosis.

Of all reported patients the majority (*n* = 317, 36%) was treated with anti-CD20 therapies, either ocrelizumab or rituximab. Another 100 patients (12%) received interferons or glatiramer acetate. They were summarized under the term “injectables”. Of the reported MS patients (*n* = 97), 11% were not treated with DMT. The remaining patients were distributed relatively evenly among the natalizumab, fingolimod, dimethyl-fumarate and teriflunomide groups (Figure 1). Relatively few cases of COVID-19 patients on cladribine (*n* = 29) or alemtuzumab therapy (*n* = 12) were reported.

### 3.2. Outcome of SARS-CoV-2 Positive MS Patients

**Figure 2:**
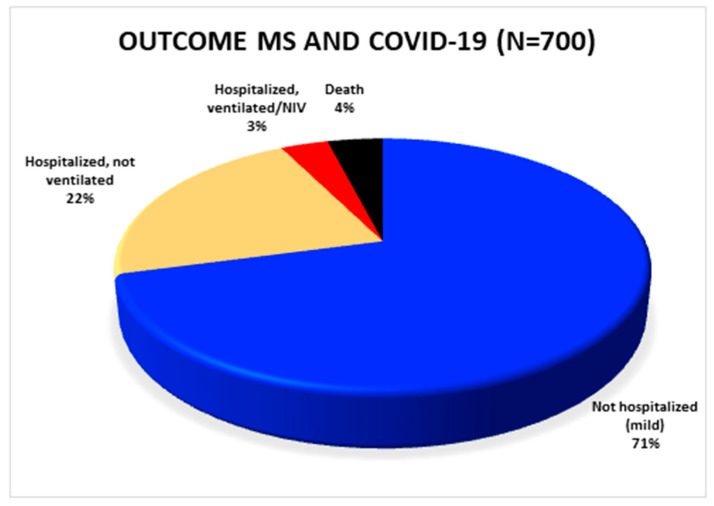
Overview of the outcome of all published SARS-CoV-2 positive MS patients in whom the outcome is known. COVID-19: coronavirus disease 2019; MS: multiple sclerosis.

Out of a total of 873 patients, published, detailed clinical outcome data were available for 700 patients (Figure 2). A total of 204 patients was hospitalized. Of these, 24 patients required non-invasive or invasive ventilation. Considering the 700 patients with known outcome, 4% (*n* = 28) of these patients died in total. The remaining 496 subjects were able to stay at home after SARS-CoV-2 infection had been diagnosed.

### 3.3. Outcome of SARS-CoV-2 Positive MS Patients in Association with the Respective DMTs

**Figure 3:**
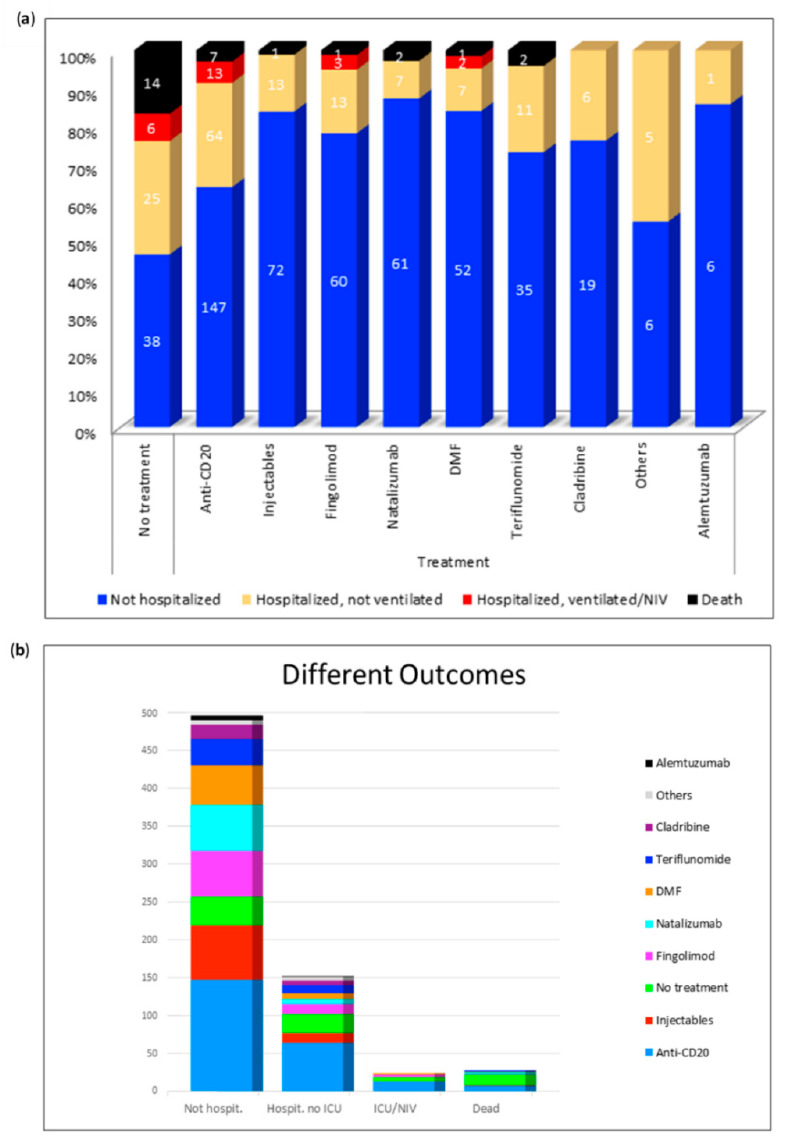
Distribution of the respective outcomes depending on the disease modifying therapies. (**a**) Distribution of different outcomes per DMT; (**b**) Number of non-hospitalized, hospitalized, ventilated, and deceased patients. DMF: dimethyl-fumarate; Hospit.: hospitalized; ICU: intensive care unit; NIV: non-invasive ventilation. Others: stem cell therapy (*n* = 5), immunoglobulins (*n* = 4), mycophenolate mofetil (*n* = 3), azathioprine (*n* = 1), cyclophosphamide (*n* = 1), methotrexate (*n* = 1).

Figure 3 gives an overview of all the different DMTs. It shows the number of non-hospitalized, hospitalized, ventilated and deceased patients under each therapy. Of all deaths (*n* = 14), 50% occurred in untreated patients. We can conclude from the figure that individual patients treated with anti-CD20-therapies, natalizumab, injectables, DMF, fingolimod, or teriflunomide died of COVID-19. In each DMT group there were hospitalized patients, although they rarely required ventilation. By far the majority of the published patients were treated with anti-CD20 therapy. Of these, 147 patients (64%) did not require hospital treatment, 64 (28%) were hospitalized but did not require ventilation, 13 patients (6%) were ventilated and a total of 7 patients (3%) out of 231 died. In the literature 83 SARS-CoV-2 positive MS patients without DMTs are found. Of these patients 14 (17%) died and a total of 31 patients (37%) were treated in hospital. None of the subjects treated with alemtuzumab or cladribine had a severe course of the disease. The hospitalization rates were 24% (cladribine) and 14% (alemtuzumab), respectively. With regard to the other DMTs, a very similar number of severe and fatal courses could be observed. Only two patients (natalizumab, teriflunomide) or one patient (fingolimod, dimethyl-fumarate, injectable) of all published cases died. Thus, the death rates were 4% (teriflunomide), 3% (natalizumab), 2% (dimethyl-fumarate), and 1% (fingolimod and injectable). Non-fatal severe courses requiring ventilation were only described for fingolimod (*n* = 3; 4%) and dimethyl-fumarate (*n* = 2; 3%).

### 3.4. Characteristics of Deceased Patients

Table 1 contains detailed information on all deceased MS patients published to date. Twenty-eight of the described SARS-CoV2 positive MS patients died. Of these, 14 patients had no DMT and 7 received anti-CD20 treatment. Of the patients in whom the course of MS was known, 14 patients (64%) had a primary or secondary progressive form and only eight patients (36%) were diagnosed with relapsing MS. The median duration of the disease was 24 years (range 5–51 years) and the median EDSS value amounted to 6.5 (range 1.5–9.5). Information on the gender and age of the patients is unfortunately scarce. However, it is generally apparent that the deceased patients were on average of older age. Twelve (43%) of those who died from COVID-19 had at least one cardiovascular comorbidity, nine of whom were significantly overweight. Two patients were diagnosed with asthma or chronic obstructive pulmonary disease and nine further patients had other concomitant diseases, including four cases of malignant disease. Only five (18%) of the deceased patients had no pre-existing conditions other than MS.

### 3.5. Analysis of Detailed Case Reports

A total of 51 SARS-CoV-2 positive MS patients had been published in detailed case reports or small case series (Supplementary Table S1). The reported patients had a median age of 45 years (min. 18, max. 62 years). Their median Expanded Disability Status Scale (EDSS) was 3.5, ranging between 0 and 8.0. Most of them had a longer disease duration of up to 28 years; the mean disease duration was 11 years. Regarding the DMTs, 16 patients were receiving ocrelizumab, nine were treated with fingolimod and eight patients were under teriflunomide therapy. The remaining patients received the following therapies: alemtuzumab (*n* = 5), cladribine (*n* = 4), natalizumab (*n* = 4), rituximab (*n* = 4), and interferon beta 1-a (*n* = 1).

For a total of nine patients, cardiovascular comorbidities such as arterial hypertension, dyslipidemia, obesity, or diabetes mellitus had been reported. Two patients either suffered from asthma or COPD and another 7 patients had other comorbidities, for example breast cancer, epilepsy, or chronic migraine. The totality of the published case reports showed a quite favorable outcome: twenty-two patients were able to stay at home, and 25 patients had to be hospitalized, but did not require non-invasive or invasive ventilation. Only three patients had to be treated in the intensive care unit including intubation or non-invasive ventilation and one patient died from COVID-19. Two of the four patients with a severe or fatal disease course suffered from cardiovascular comorbidities.

## 4. Discussion

The SARS-CoV-2 pandemic is a threat to many billions of people, many of whom are chronically ill. As neurologists, we are particularly interested in assessing the risk for those patients whose immune system is impaired by drug therapy. When we summarize the data of the literature regarding MS therapeutics and their potential risk in the context of the pandemic, it must be remembered that there is a certain publication bias. In general, more results are published for the highly effective and more innovative therapies, such as anti-CD20 treatment, than for the platform therapies. However, the numbers of serious or fatal cases remain surprisingly low overall. Considering all published cases of MS patients with COVID-19, the rate of fatal outcome is 4%. Even in the group of patients with anti-CD20 therapies, the rate of reported deaths is 3%. The proportion of patients undergoing intensive care is 6%. Similar or even better outcome results have been reported for patients treated with other MS therapeutics. It is noticeable that the group of untreated MS patients falls off quite markedly. Of the 83 people for whom the outcome was published, 17% died from COVID-19 and another 7% needed non-invasive or mechanical ventilation. These results might indicate that the MS therapy itself could even be protective or that the patients without immunomodulating therapy rather belong to the group of progressive MS patients and thus are more likely to show an advanced stage of the disease [18]. This suggestion is in line with results of Parotta and colleagues who identified older age, presence of comorbidities, progressive disease, and a non-ambulatory status, but not DMTs as risk factors for COVID-19 critical illness or related death [19]. Similar results can be seen in Table 1, as the majority of deceased patients have (cardiovascular) comorbidities, a progressive course of MS and a high degree of disability. Considering that the hyperactivity of the immune system as a consequence of the SARS-CoV-2 infection may cause even greater damage [72,73,74], DMTs could possibly avert a severe disease course. Lung tissue is especially affected by the unleashed immune factors. Thus, COVID-19 associated hyperinflammation can ultimately lead to ARDS [75,76,77]. Kloc et al. show that the application of small GTPase RhoA pathway inhibitors prevents macrophage infiltration and inhibits lung inflammation [78]. The MS therapeutics fingolimod and Siponimod are able to inhibit RhoA and RhoA/actin-dependent macrophage receptors recycling, and expression, and might be evaluated as a potential ARDS protective treatment [78].

In addition to age, cardiovascular comorbidities have been identified as important risk factors for severe COVID-19 in recent studies [79,80,81]. Interestingly, obesity in particular appears to be a decisive risk factor. The fact that obesity can reduce immune cell functionality, induce gut microbiome imbalance, as well as an inflammatory cytokine phenotype, may be a possible explanation for the correlation between obesity and severe COVID-19 course [82]. In summary, there is strong evidence that the suggested risk factors and the disability of MS have a significantly greater impact on the risk of developing serious COVID-19 disease than treatment with different DMTs. In one particular case, a young MS patient showed a mild course of COVID-19 even a few days after an alemtuzumab infusion [23]. This example emphasizes the fact that, even during treatment with highly effective DMTs, severe disease progression is not necessarily to be expected. However, especially for anti-CD20 therapies, the risk profile needs to be further explored. Very similar observations were also made in patients treated with disease-modifying anti-rheumatic drugs (DMARDs) due to inflammatory bowel disease or rheumatoid diseases [83,84,85].

This work has some limitations: First, this is a retrospective work that does not allow for causal conclusions. Secondly, we only describe the results published so far, but further analyses including multivariate logistic regression models have not been made and would exceed the scope of this paper. Finally, the already mentioned publication bias must be taken into account.

## 5. Conclusions

In conclusion, it is important to address the risks of different patient groups in the context of the SARS-CoV-2 pandemic. It is reasonable to expect that patients with chronic autoimmune diseases such as MS, who often require immunosuppressive therapies, will be at a particularly high risk for severe COVID-19 course. However, the cases currently published in the literature show that even among these patients the death rate is comparatively low and that it is not the DMTs themselves that pose a risk; rather, age, co-morbidities and the severity of the underlying disease are much more important. The fact that patients without DMTs account for a significant proportion of all deaths suggests that DMTs may even be protective.

## Figures and Tables

**Table 1:** Characteristics of deceased SARS-CoV-2 positive MS patients. BMI: body-mass-index; COPD: chronic obstructive pulmonary disease; DMT: disease modifying therapy; EDSS: Expanded Disability Status Scale; f: female; m: male; N/A: not available; PPMS: primary progressive multiple sclerosis; RRMS: relapsing-remitting multiple sclerosis; SPMS: secondary progressive multiple sclerosis; VTE: venous thromboembolism.

Pat. ID	Sex (m/f)	Age	Disease Duration (years)	Disease Phase	EDSS	DMT	Comorbidities
01	N/A	6th decade	25	SPMS	7.5	None	None
02	N/A	8th decade	51	SPMS	8	None	Chronic myelomonocytic leukemia
03	N/A	6th decade	28	SPMS	8.5	None	Ischemic heart disease, COPD
04	N/A	8th decade	22	PPMS	8.5	None	None
05	N/A	6th decade	48	SPMS	9	None	Colorectal cancer
06	N/A	7th decade	35	SPMS	9	None	Arterial hypertension
07	N/A	4th decade	28	SPMS	9.5	None	None
08	N/A	N/A	N/A	N/A	4	None	Obesity
09	N/A	N/A	N/A	N/A	7	None	Asthma
10	f	50	13	RRMS	N/A	None	Obesity, hypertension, hypothyroidism
11	f	65	31	SPMS	N/A	None	Intrathecal baclofen pump
12	N/A	5th decade	23	RRMS	1.5	None	Obesity
13	N/A	7th decade	47	RRMS	5	None	None
14	m	74	N/A	SPMS	8.5	None	Coronary artery disease, hypertension, diabetes mellitus, COPD, cardiomyopathy
15	N/A	5th decade	22	PPMS	7	Rituximab	None
16	N/A	N/A	N/A	N/A	N/A	Rituximab	Sjögren’s syndrome, hypothyroidism
17	N/A	N/A	N/A	N/A	N/A	Rituximab	Obesity
18	f	43	18	SPMS	6.5	Rituximab	Hypothyroidism
19	m	42	18	RRMS	N/A	Rituximab	Hodgkin lymphoma, anticoagulation for VTE, intrathecal baclofen pump
20	m	66	33	SPMS	N/A	Ocrelizumab	History of testicular and prostate cancer, intrathecal baclofen pump
21	N/A	N/A	N/A	N/A	6	Ocrelizumab	COPD
22	f	60	19	RRMS	N/A	Natalizumab	Obesity, coronary artery disease, hypertension
23	f	51	14	RRMS	6.5	Natalizumab	Obesity, hypertension, recurrent urinary tract infections
24	f	55	N/A	SPMS	7.5	Teriflunomide	Myotonic dystrophy
25	N/A	3th decade	5	RRMS	3	Teriflunomide	Obesity
26	N/A	N/A	N/A	N/A	N/A	Fingolimod	Severe cognitive impairment
27	N/A	5th decade	5	RRMS	3	Dimethyl-fumarate	Obesity, grade 2 lymphopenia, schizophrenia
28	m	71	30	SPMS	N/A	Glatiramer-acetate	Obesity, anticoagulation for VTE

## Supplementary Materials

The following are available online at https://www.mdpi.com/2077-0383/9/12/4067/s1, Table S1: Detailed overview of published case reports on SARS-CoV-2 positive MS patients. ARDS: acute respiratory distress syndrome; COPD: chronic obstructive pulmonary disease; COVID-19: coronavirus disease 2019; DMF: dimethyl-fumarate; DMT: disease modifying therapy; EDSS: Expanded Disability Status Scale; f: female; m: male; MS: multiple sclerosis; N/A: not applicable; PPMS: primary progressive multiple sclerosis; RRMS: relapsing-remitting multiple sclerosis; SPMS: secondary progressive multiple sclerosis.
