# Supplementary material for: Experience in Multiple Sclerosis Patients with COVID-19 and Disease-Modifying Therapies: A Review of 873 Published Cases
Source: J Clin Med. 2020 Dec 16;9(12):4067. doi: 10.3390/jcm9124067 (PMC7766122; doi:10.3390/jcm9124067)
Supplement: Supplementary file 1 [file jcm-09-04067-s001.pdf]

Paper, author	Sex (m/f)	Age (years)	RRMS/PPMS /SPMS	Actual EDSS	Duration MS (years)	Recent MS therapy (duration)	Previous MS therapies	relevant comorbidities (esp. Cardiovascular risk factors)	COVID-19 symptoms	COVID drug therapy	Outcome COVID-19
Aguirre et al, Multiple Sclerosis and related disorders	m	18	RRMS	1.5	7	Natalizumab (6 months)	Interferon beta 1-a, DMF	None	Fever, cough, malaise	Hydroxychloroquine, antibiotics	Hospitalized for 1 week, discharged without residual symptoms
Arca et al, SN comprehensive clinical medicine	f	58	N/A	N/A	N/A	Fingolimod		Chronic migraine (fremanezumab therapy)	Cough, fever, generalized weakness, headache, shortness of breath	Hydroxychloroquine, antibiotics	**Intensive care unit treatment, intubation**, complete recovery
Barzegar et al, Neurol Neuroimmunol Neuroinflamm	f	42	RRMS	1.0	19	Fingolimod (4 years)	Methotrexate, interferon beta 1-a	Hypothyroidism, recurrant urinary tract infection, history of pulmonary embolism	Dry cough, dyspnea, fever, tachycardia, increased respiratory rate	Hydroxychloroquine	Hospitalized for 13 days, discharged without residual symptoms
Bollo et al, J Neurological Scienes	f	34	RRMS	2.5	4	Fingolimod (3 years)	DMF	N/A	Fever, pharyngeal pain	Hydroxychloroquine, lopinavir/ritonavir	Hospitalized, discharged without residual symptoms
	f	58	RRMS	2.5	5	Teriflunomide (4 years)	Interferon beta 1-a	N/A	Fever, diarrhea	Hydroxychloroquine, lopinavir/ritonavir, antibiotics	Hospitalized, need for oxygen support, discharged without residual symptoms
Borriello & Ianniello, Multiple Sclerosis and related disorders	m	28	RRMS	1.5	20	Natalizumab (21 months)	DMF	None	Fever, dyspnea	Hydroxychloroquine, antipyretics, antibiotics	Hospitalized for 10 days, discharged without residual symptoms
Carandini et al, Multiple Sclerosis Journal	f	25	RRMS	N/A	1	Alemtuzumab (1 year)	None	None	Dry cough, fatigue, fever	None	Not hospitalized, mild symptoms, complete recovery
Celius, Multiple Sclerosis and related disorders	f	35	RRMS	N/A	2	Cladribine (1 year)	Fingolimod	None	Anosmia, slight fever	None	Not hospitalized, mild symptoms, complete recovery
Chiarini et al, Journal of Neuroimmunology	f	45	RRMS	6.5	24	Fingolimod (3 years)	Interferon beta 1-a, glatiramer acetate, mitoxantrone, teriflunomide	**Obesity**	Fever, asthenia	Hydroxychloroquine, lopinavir/ritonavir, paracetamol	Hospitalized for 15 days, need for oxygen support, discharged without residual symptoms
Ciardi et al, Journal of Neuroimmunology	f	62	RRMS	6.0	N/A	Teriflunomide (5 years)	N/A	N/A	Fever, dyspnea, severe fatigue, dry cough, diarrhea	Hydroxychloroquine, lopinavir/ritonavir	Hospitalized, discharged without residual symptoms
Conte, Multiple Sclerosis and related disorders	f	48	N/A	N/A	N/A	Ocrelizumab	N/A	N/A	Fever, upper respiratory symptoms, malaise	Hydroxychloroquine, antibiotics	Hospitalized for 3 days, prolonged symptomatic period (7 weeks)
De Angelis et al, Multiple Sclerosis and related disorders	m	29	RRMS	1.5	2	Cladribine (19 months)	None	None	Anosmia, dysgeusia, diahhrea, fever	None	Not hospitalized, mild symptoms, complete recovery
	f	61	RRMS	2.5	26	Cladribine (17 months)	Interferon beta 1-a, fingolimod	**Hypertension, dyslipidemia**	No symptoms	None	Not hospitalized, no symptoms
Dersch et al, Multiple Sclerosis Journal	m	59	RRMS	N/A	4	Cladribine (2 weeks)	DMF	N/A	Fever, malaise, progressive respiratory symptoms	Hydroxychloroquine, antibiotics, aviptadil	Hospitalized for 16 days, discharged without residual symptoms
Devogelaere et al, Neurological Sciences	f	33	RRMS	8.0	16	Rituximab (6 months)	Interferon beta 1-a	N/A	Fever, muscle soreness, headache, cough, mild dyspnea	None	Hospitalized, discharged without residual symptoms
Fernandez-Diaz et al, Multiple Sclerosis and related disorders	m	43	RRMS	0	N/A	Alemtuzumab	N/A	None	Fever, cough, myalgia, shortness of breath	Hydroxychloroquine, lopinavir/ritonavir, antibiotics	Not hospitalized, mild symptoms, complete recovery
	f	30	RRMS	2.5	N/A	Alemtuzumab (1 year)	None	None	Fever, cough, dyspnea	Hydroxychloroquine, antibiotics	Hospitalized, discharged without residual symptoms
Celsi&Lorna, Multiple Sclerosis and related disorder	f	24	RRMS	0	1.5	Alemtuzumab (15 months)	None	N/A	Cough, myalgia	None	Not hospitalized, mild symptoms, complete recovery
Foerch et al, Multiple Sclerosis and related disorders	f	57	RRMS	2.0	10	Fingolimod (9 years)	None	N/A	Fever, cough, low peripheral oxygen saturation	None	**Intensive care unit treatment, non-invasive ventilation,** complete recovery

Gemcioglu et al, Multiple Sclerosis and related disorders	m	31	N/A	N/A	2	interferon beta 1-a (2 years)	None	None	Cough, shortness of breath	Hydroxychloroquine, antibiotics	Hospitalized for 5 days, discharged without residual symptoms
Ghajarzadeh et al, Multiple Sclerosis and related disorders	f	39	RRMS	2.0	10	Ocrelizumab (3 months)	Interferon beta 1-a	Epilepsy	Fever, mild dyspnea	None	Not hospitalized, mild symptoms, complete recovery
Gomez-Mayordomo et al,	m	57	RRMS	6.0	24	Fingolimod (7 years)	Interferon beta 1-a	N/A	Fever, malaise	Hydroxychloroquine	Hospitalized, delayed clinical exacerbation of COVID-1, discharged without residual symptoms
Guevara et al, Multiple Sclerosis and related disorders	m	35	RRMS	N/A	1.5	Alemtuzumab (1.5 years)	None	None	Mild fever, cough	None	Not hospitalized, mild symptoms, complete recovery
Iannetta et al, Multiple Sclerosis and related disorders	f	36	RRMS	5.5	2	Ocrelizumab (1 year)	N/A	Papillary thyroid carcinoma	Cough, coryza	None	Hospitalized, discharged without residual symptoms
	m	54	SPMS	7.0	17	Ocrelizumab (1.5 year)	Interferon beta 1-a, fingolimod	Deep venous thrombosis	Fever	None	Hospitalized, discharged without residual symptoms
Louapre et al, Revue neurologique	f	N/A	SPMS	6.0	28	Ocrelizumab (2 weeks)	Interferon beta 1-a, fingolimod, natalizumab	**Hypertension, dyslipidemia**	No symptoms	None	Not hospitalized, no symptoms
	f	56	RRMS	8.0	21	Natalizumab (13 yers)	Interferon beta 1-a, glatiramer acetate	**COPD**, epilepsy	Fever, cough	N/A	Hospitalized, discharged without residual symptoms
Lucchini et al, Multiple Sclerosis and related disorders	f	60	RRMS	2.5	8	Ocrelizumab (1 year)	Glatiramer acetate, DMF, fingolimod	N/A	Fever, cough	Antibiotics, prednisone	Not hospitalized, mild symptoms, complete recovery
Maghzi et al, Journal of Neurology	m	52	RIS	0	3	Teriflunomide (3 years)	None	Obstructive sleep apnea, **hyperlipidemia,** depression	Fatigue, headache, hyposmia, myalgia, fever, diarrhea	None	Not hospitalized, complete recovery
	f	52	RRMS	N/A	18	Teriflunomide (3.5 years)	N/A	None	Fever, headache, nausea, vomiting, hyposmia	None	Not hospitalized, complete recovery
	m	47	RRMS	N/A	5	Teriflunomide (4 years)	Interferon beta 1-a	None	Diarrhea, cough, dyspnea		Not hospitalized, mild symptoms, complete recovery
	m	38	RRMS	N/A	11	Teriflunomide (5 months)	Glatiramer acetate	Alcohol abuse, homelessness	Fever, cough	None	Not hospitalized, mild symptoms, complete recovery
	f	79	SPMS		28	Teriflunomide (2.5 years)	Interferon beta 1-a	**Hypertension**, recurrent urinary tract infections	Sore throat	N/A	Hospitalized, discharged without residual symptoms
Mallucci et al, Multiple Sclerosis and related disorders	N/A	37	RRMS	2.5	14	Fingolimod (9 years)	Interferon beta 1-a, natalizumab	N/A	No symptoms	None	Not hospitalized, no symptoms
	N/A	49	RRMS	2.5	19	Fingolimod (10 months)	Interferon beta 1-a, glatiramer acetate	N/A	No symptoms	None	Not hospitalized, no symptoms
Meca-Lallana et al, Multiple Sclerosis and related disorders	m	60	RRMS	8.0	10	Rituximab (3 years)	Glatiramer acetate, natalizumab	N/A	Fever, cough, dyspnea	Hydroxychloroquine	Hospitalized for 5 days, discharged without residual symptoms
	m	49	RRMS	3.0	6	Ocrelizumab (3 years)	Glatiramer acetate	**Smoking**	Fever, cough, dyspnea	Lopinavir/ritonavir	Hospitalized for 5 days, discharged without residual symptoms
	m	45	RRMS	2.0	5	Ocrelizumab (3 years)	Teriflunomide	N/A	Fever, cough, dyspnea, myalgia	Hydroxychloroquine, lopinavir/ritonavir, antibiotics	Hospitalized for 4 days, discharged without residual symptoms
	f	25	RRMS	1.0	8	Ocrelizumab (1 month)	Natalizumab, fingolimod, rituximab	N/A	No symptoms	None	Not hospitalized, no symptoms
	f	36	RRMS	2.0	11	Ocrelizumab (8 months)	Glatiramer acetate, rituximab	N/A	Fever, headache	None	Not hospitalized, complete recovery
	f	60	PPMS	7.5	16	Ocrelizumab (9 years)	None	N/A	Fever, cough, headache	None	Not hospitalized, complete recovery
	m	52	RRMS	N/A	11	Ocrelizumab (1 year)	Glatiramer acetate	N/A	No symptoms	None	Not hospitalized, no symptoms

Möhn et al, Journal of Neurology	m	42	RRMS	0	7	Teriflunomide (7 years), methylprednisolone 1g/d, 4 days)	None	None	Nausea, vomiting, general weakness, fever, hypotension, tachycardia	None	Hospitalized, discharged without residual symptoms
Novi et al, Multiple Sclerosis and related disorders	m	58	PPMS	6.0	11	Ocrelizumab (2 years)	Interferon beta 1-a, glatiramer acetate, fingolimod	**Asthma**	Fever, cough	None	Hospitalized for 2 days, discharged without residual symptoms
Rimmer et al, Multiple Sclerosis Journal	f	51	RRMS	6.5	8	Natalizumab (7 years)	Interferon beta 1-a	**Obesity, hypertension,** recurrent urinary tract infections	Fever, cough, dyspnea, ARDS	Hydroxychloroquine, antibiotics	Intensive care unitvtreatment, intubation + vasopressors, **death 12 days after hospitalization**
Suwanwongse & Shabarek, Multiple Sclerosis and related disorders	m	31	N/A	N/A	N/A	Ocrelizumab (2 years)	N/A	**Obesity**	Cough, dyspnea, nausea, vomiting, diarrhea	Hydroxychloroquine	Hospitalized for 5 days, discharged without residual symptoms
Rempe Thornton & Harel, Multiple Sclerosis and related disorders	m	42	RRMS	N/A	4	Ocrelizumab (9 months)	N/A	None	Fever, cough, impaired taste, dyspnea	None	Not hospitalized, dysgeusia persisted > 1 month
	f	39	RRMS	N/A	5	Ocrelizumab (4 months)	N/A	None	Cough	None	Not hospitalized, mild symptoms, complete recovery
Valencia-Sanchez & Wingerchuk, Multiple Sclerosis and related disorders	f	58	RRMS	6.0	13	Fingolimod (9 years)	Interferon beta 1-a, glatiramer acetate, natalizumab	**Diabetes mellitus, hypertension, obesity, TIA**	Fever, cough, dyspnea	Hydroxychloroquine, antibiotics, tocilizumab	**Intensive care unit treatment, intubation, vasopressors,** residual hyposmia and dysgeusia
Woo et al, Journal of Neurology	f	44	RRMS	2.0	21	Rituximab (7 years)	N/A	Breast carcinoma	Fever, cough, malaise, myalgia, dyspnea	None	Hospitalized for 4 days, discharged without residual symptoms
Wurm et al, Multiple Sclerosis Journal	f	59	N/A	6.0	4	Rituximab (2.5 years)	N/A	N/A	Fever, cough, dyspnea, headache, nausea	Antibiotics	Hospitalized for 13 days, discharged without residual symptoms

Supplementary Table 1: Detailed overview of published case reports on SARS-CoV-2 positive MS patients. ARDS: acute respiratory distress syndrome; COPD: chronic obstructive pulmonary disease; COVID-19: coronavirus disease 2019; DMF: dimethylfumarate; DMT: disease modifying therapy; EDSS: Expanded Disability Status Scale; f: female; m: male; MS: multiple sclerosis; N/A: not applicable; PPMS: primary progressive multiple sclerosis; RRMS: relapsing-remitting multiple sclerosis; SPMS: secondary progressive multiple sclerosis;
